# Wobble Editing of *Cre*-box by Unspecific CRISPR/Cas9 Causes CCR Release and Phenotypic Changes in *Bacillus pumilus*

**DOI:** 10.3389/fchem.2021.717609

**Published:** 2021-08-09

**Authors:** Yingxiang Wang, Linfeng Cao, Meiying Bi, Sicheng Wang, Meiting Chen, Xingyu Chen, Ming Ying, Lei Huang

**Affiliations:** ^1^ Tianjin Key Laboratory of Organic Solar Cells and Photochemical Conversion, School of Chemistry and Chemical Engineering, Tianjin University of Technology, Tianjin, China; ^2^ Tianjin Key Laboratory of Drug Targeting and Bioimaging, School of Chemistry and Chemical Engineering, Tianjin University of Technology, Tianjin, China

**Keywords:** *Bacillus pumilus*, UgRNA:Cas9, cre-box, CCR, multiple gene wobble editing

## Abstract

CRISPR-associated Cas9 endonuclease (CRISPR/Cas9) systems are widely used to introduce precise mutations, such as knocking in/out at targeted genomic sites. Herein, we successfully disrupted the transcription of multiple genes in *Bacillus pumilus* LG3145 using a series of unspecific guide RNAs (gRNAs) and UgRNA:Cas9 system-assisted *cre*-box editing. The bases used as gRNAs shared 30–70% similarity with a consensus sequence, a *cis-*acting element (*cre*-box) mediating carbon catabolite repression (CCR) of many genes in *Bacillus*. This triggers *trans*-crRNA:Cas9 complex wobble cleavage up/downstream of *cre* sites in the promoters of multiple genes (up to 7), as confirmed by Sanger sequencing and next-generation sequencing (NGS). LG3145 displayed an obvious CCR release phenotype, including numerous secondary metabolites released into the culture broth, ∼ 1.67 g/L white flocculent protein, pigment overflow causing orange-coloured broth (absorbance = 309 nm), polysaccharide capsules appearing outside cells, improved sugar tolerance, and a two-fold increase in cell density. We assessed the relationship between carbon catabolite pathways and phenotype changes caused by unspecific UgRNA-directed *cre* site wobble editing. We propose a novel strategy for editing consensus targets at operator sequences that mediates transcriptional regulation in bacteria.

## Introduction

*Bacillus pumilus*, a Gram-positive soil bacterium, is considered a potential host strain for the production of chemicals, heterologous proteins and antimicrobial materials (Westers et al., 2004; Su et al., 2020). However, in *Bacillus*, there is a general regulatory mechanism regulating many catabolic genes and operons through repression by favoured carbon sources such as glucose, fructose or mannose, termed carbon catabolite repression (CCR) (Stülke and Hillen, 2000). CCR helps to maintain carbon catabolic/metabolic network balance according to metabolic capacities, and can act as a serious barrier preventing the use of *Bacillus* as cell factories. Thus, alleviating CCR is an established area of research (Chen et al., 2020). Three common regulatory components have been identified that silence multiple operons: the catabolite responsive element *cre*-box, a promoter proximal *cis*-acting conserved sequence that negatively regulates multiple genes in *Bacillus* species; the histidine phosphocarrier protein Hpr, an intermediate of the phosphoenolpyruvate:sugar transport system (PTS); and the catabolite control protein CcpA, a *trans*-acting factor that binds *cre*-box through Hpr-Ser-p to control target gene transcription (Peng et al., 2020; Langa et al., 2021; Neira et al., 2021).

Accumulating evidence indicates that *cre*-boxes are globally distributed in the genome of *Bacillus*, hence simultaneous point mutations at these *cre*-boxes may trigger global regulation network rearrangement (Zhang et al., 2018; Han et al., 2020). In the present study, we wished to manipulate this trigger in *Bacillus pumilus* to disrupt the transcriptional regulation of multiple genes simultaneously to overcome CCR limitations, enhance sugar resistance, and improve secretion. The clustered regularly interspaced short palindromic repeats (CRISPR)/Cas9 system, a primitive adaptive immunity system in bacteria that defends against foreign genetic contamination, is capable of editing multiple genes simultaneously (Jinek et al., 2012; Cong et al., 2013). Specific editing using the CRISPR/Cas9 system cleaves target sites in the genome, but the approach suffers from genome-wide off-target effects (Guilinger et al., 2014; Wu et al., 2019). However, the off-target effects may be utilised to edit the consensus sequence at multiple-gene loci such as *cre*-boxes. Thus, an unspecific CRISPR/Cas9 system was constructed to control guide RNA (gRNA):Cas9 complex off-target effects. Specifically, three 20 nt oligonucleotides sharing 30–70% similarity with target sites were synthesised to serve as single gRNAs involved in targeting seven genes (listed in Supplementary Table S1).

Herein, we used a pCas9 derivate plasmid to assist multiple *cre*-box editing. The plasmid encodes Cas9 nuclease and a double-stranded DNA (dsDNA) endonuclease that uses a CRISPR RNA (crRNA) guide to specify the site of cleavage (Jiang et al., 2013). Spacers (gRNA) can be inserted into the crRNA array between *Bsa*I sites using annealed oligonucleotides. We reprogramed three unspecific gRNAs sharing 30–70% similarity with the target fragments (as shown in Supplementary Table S2) and reconstructed three plasmids (pCas9-*Cax*, pCas9-*Cac* and pCas9-*Cpt*). Simultaneous use of three unspecific gRNAs enabled wobble editing up/downstream of *cre*-box sites in the seven target genes. Sanger sequencing and next-generation sequencing (NGS) were used to confirm mutagenesis near the *cre*-boxes (-10 and -35 domains falling in the range of ± 100 bp). The unspecific CRISPR/Cas9 system led to pleiotropic genome mutations in *B. pumilus*, resulting in strain LG3145, which exhibited remarkable phenotypic changes, such as smoother colonies, altered colour pigments, polysaccharide capsules outside cells, and elevated secretion. It is possible that CCR release of numerous genes may be responsible for the observed phenotypical changes.

## Materials and Methods

### Strains and Reagents

Previously, we isolated a *B. pumilus* wild-type strain from soil, and the genotype was the same as that of *B. pumilus* SH-B9 (NCBI Accession no: NZ_CP011007.1) according to whole-genome sequencing. The other strains used in this study are listed in Supplementary Table S3. *Escherichia coli* DH5α (the host for pCas9) and *Escherichia coli* Top10, both purchased from Thermo Fisher Scientific (Waltham, MA, United States), were used for plasmid construction and amplification, and cultured in Luria-Bertani medium containing 25 μg/ml chloromycetin. All chemicals, a DNA gel purification kit, and a plasmid extraction kit were purchased from TIANGEN Biotech (Beijing, China). Restriction enzymes and DNA ligase were purchased from New England Biolabs (Ipswich, MA, United States). Taq DNA polymerase and oligonucleotides used for PCR experiments were purchased from Ruibio Biotech (Beijing, China).

### Plasmids and Primers

**FIGURE 1:**
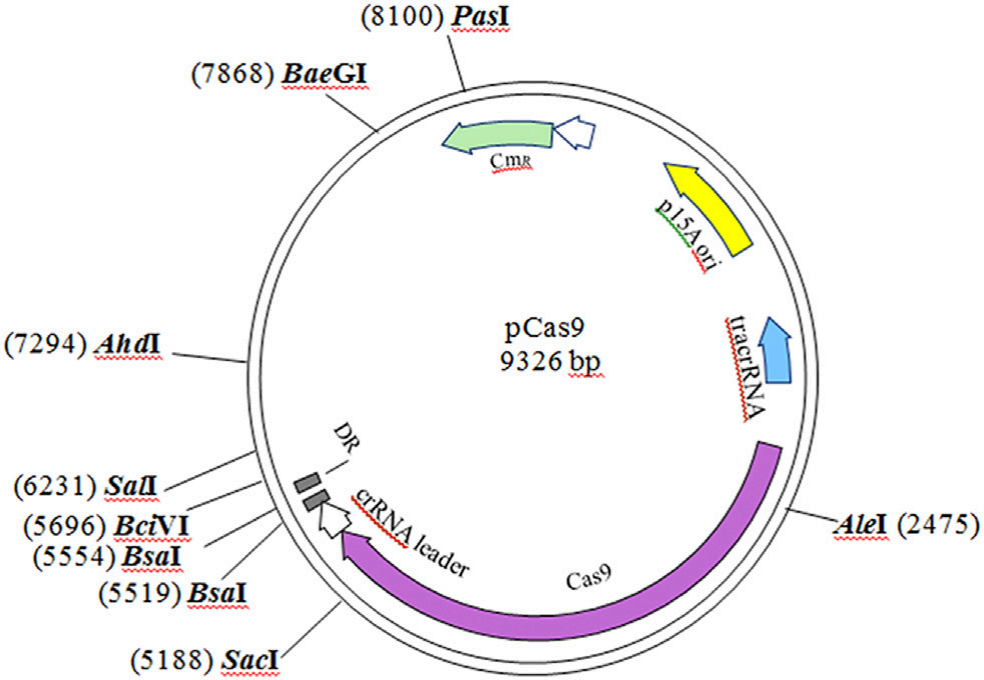
The original pCas9 plasmid used in this experiment.

The pCas9 plasmid and sequences used in this study (Supplementary Table S4) are available from the Addgene nonprofit plasmid repository (Cat: 42,876). The pCas9 plasmid is 9,326 bp based on the low-copy backbone of pACYC184 (ATCC 37033), which endows resistance to chloramphenicol (25 μg/ml). The other elements of this plasmid include tracrRNA, Cas9, and an array of crRNAs with two *Bsa*I sites for spacers (Figure 1). The plasmids pCas9-*Cax*, pCas9-*Cac* and pCas9-*Cpt* were constructed for *cre-*box editing of seven target genes. The spacer oligonucleotides for cloning into pCas9 and the primers used for PCR are listed in Supplementary Table S5.

### Unspecific gRNA Array Construction and Multiple *cre* Sites Editing Strategy

For editing multiple *cre*-box sites at the seven operons and simultaneously controlling the range of gRNA:Cas9 complex off-targets, oligonucleotides of gRNAs must direct the Cas9 protein to recognize multiple target sites. Because the *cre*-boxes of *B. pumilus* have the consensus sequence WWGNAANCGNWNNCW (Supplementary Table S1), we chose NNG for the PAM and *cre* proximal sequences for target sequences designed using the Zhang Lab website (http://crispor.tefor.net/), and the results (Supplementary Table S2) include partial or complete *cre* sequences (Supplementary Figure S2). In order to better describe the seven target operons, we continued using the same gene symbols of the type strain *B. subtilis*168 (NCBI Accession no: NC_000964.3) where annotation was consistent. The seven target sequences were divided into three groups with the highest (70%), moderate (70–50%), and lowest (50–30%) identity with the three unspecific gRNAs (*Cac*, *Cpt*, and *Cax*).

**FIGURE 2:**
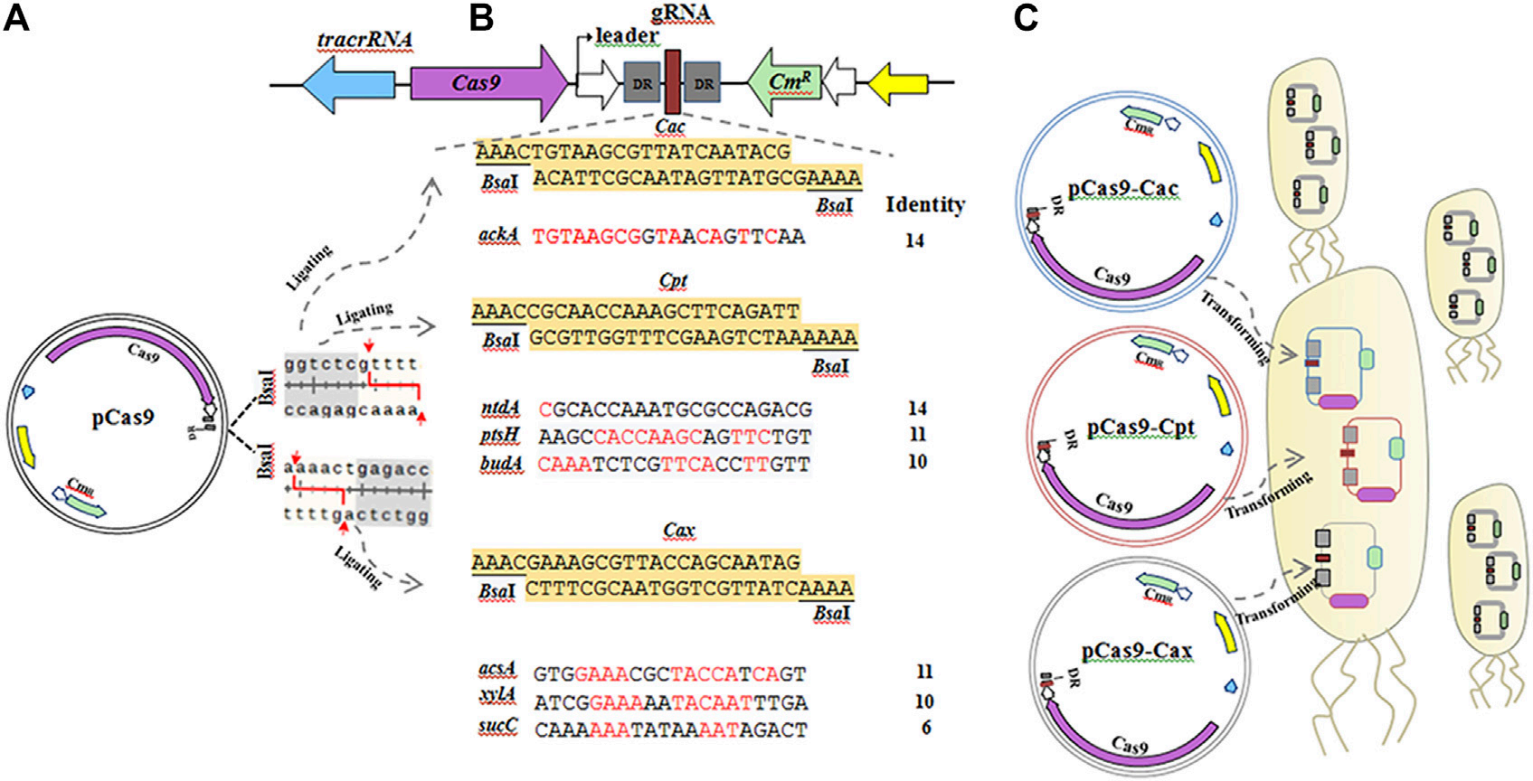
The strategy for multiple editing at *cre* sites in seven operons using three unspecific gRNA:Cas9 systems in *B. pumilus*. **(A)** The double *Bsa*I restriction sites of pCas9 allow the gRNA to be inserted. **(B)** The three unspecific gRNAs used in this study contain 24 bp oligonucleotides and two *Bsa*I sites at both ends, in which *Cax* has the highest identity (70%) with 14 bp shared with the target sequences of *ack*A, *Cac* and *Cpt* (moderate, 50–70%), or lower identity gRNAs (30–55%) with 10–14 bp shared with *ntdA*, *ptsH* and *bud*A, and 6–10 bp shared with *acsA*, *xylA* and *sucC*. **(C)** After annealing, *Cax*, *Cac* and *Cpt* were inserted into pCas9 to construct three plasmids (pCas9-*Cax*, pCas9-*Cac* and pCas9-*Cpt*) using T4 DNA ligase, which were transformed into *B. pumilus* wild-type (WT) cells using the protoplast transformation method. Chloromycetin was added into the CMR media used for isolating transformants.

Among them, *ackA* and *ntdA* share the highest identity (70%) with the unique gRNA *Cac* and the common gRNA *Cpt* , both of which share >50% identity with *pts*H and *bud*A. The *ack*A encoding acetate kinase that converts pyruvate into acetate and is positively regulated by CcpA protein, *ntdA* encoding an aminotransferase responsible for converting dehydroglucose-6-phosphate to kanosamine-6-phosphate, *pts*H encoding phosphocarrier protein Hpr that is also repressed by CcpA, and *bud*A encoding acetolactate decarboxylase that converts pyruvate to acetoin and is also activated by CcpA. The target site sharing only 30% identity is the *suc*C operon encoding succinyl-CoA synthetase, which converts succinyl-CoA to succinate in the tricarboxylic acid (TCA) cycle and is negatively regulated by CcpA, and shares the gRNA *Cax* with two other operons (*acsA* and *xylA*) but they share more than 50% identity. Acetyl-CoA synthase, encoded by *acs*A, converts pyruvate into propanoate, which can be activated by CcpA and *xyl*A encoding a xylose isomerase responsible for converting D-glucose into D-fructose. The three unspecific gRNAs (*Cac*, *Cpt*, and *Cax*) were inserted into the *Bsa*I sites of pCas9 as the CRISPR spacers after annealing at 95°C for 5 min and 37°C for 30 min using T4 ligase according to the pCas9 protocol (Huang et al., 2016). The strategy of multiple editing at *cre* sites used in this study is depicted in Figure 2.

### UgRNA:Cas9 System-Assisted Multiple *cre* Sites Editing

*E. coli* Top10 cells were used to construct and amplify plasmids. Ligations of gRNA-annealed oligos and *Bsa*I-digested pCas9 were gently mixed with competent *E. coli* Top10 cells at a ratio of 1:200 and incubated on ice for 30 min, then heated at 42°C in a water bath for 90 s, and chilled for 1–2 min on ice. The cells were then incubated in preheated LB medium (150 μl) at 37°C for 45 min, then plated onto 25 μg/ml chloramphenicol selective media and incubated overnight at 37°C. The following morning, transformants were picked and incubated in LB medium. After cell growth, plasmids were extracted using an appropriate kit and the pCas9-*Cac*, pCas9-*Cpt* and pCas9-*Cax* constructs were verified by DNA sequencing.

Protoplasts of *B. pumilus* wild-type (WT) were transformed with plasmid DNA using a modified method described by Chang and Cohen (1979). A single colony of *B. pumilus* WT was inoculated into 5 ml of LB medium and cultured overnight at 37°C in a shaker (200 rpm). The overnight culture was diluted 50-fold into 5 ml of fresh LB medium and grown for 8 h at 37°C (200 rpm). Cells were harvested by centrifugation (9,000 *×* g, 10 min, 4°C). The supernatant was removed and cells were resuspended in 1 ml of SMMP solution (equal volumes of 2× SMM buffer and 4× Penassay broth). Lysozyme powder was added to 0.4 mg/ml, mixed, and incubated at 37°C for 45 min (100 rpm) to prepare protoplasts (globular appearance). Protoplasts were harvested by centrifugation (5,000 *×* g, 10 min, RT), the supernatant was poured off, and the cell pellet was resuspended gently in 0.05 ml SMMP. Plasmid DNA (0.2 μg) was mixed with 0.05 ml of protoplasts and added to a microtube containing 150 μl of polyethylene glycol (PEG) 6,000. After gentle mixing, the sample was incubated at 37°C for 2 min in a water bath shaker to allow plasmids to enter protoplasts, and 0.5 ml of SMMP was added for termination. Protoplasts were harvested by centrifuging (10,000 *×* g, 7 min) and the supernatant was discarded. SMMP (300 μl) was added and incubated for 60–90 min at 37°C in a water bath shaker (100 rpm) to allow for expression of the antibiotic resistance marker. Because there is no *B. pumilus* replication site in pCas9, the pCas9-*Cac*, pCas9-*Cpt* and pCas9-*Cax* plasmids were integrated into the genome. Cells were plated on CMR-selective regeneration medium containing chloramphenicol to select transformants. The three plasmids were successively transformed into *B. pumilus* WT three times. We engineered the *B. pumilus* LG3145 strain from eight transformants through DNA sequencing.

### Fermentation and Morphological Measurements

To analyse the effects of multiple *cre* sites editing on phenotype, WT and mutant strains were grown in high-glucose fermentation medium (GYN; 10–30% glucose, 2% yeast extract, 0.05% MgSO_4_•7H_2_O, 1% urea) in triplicate at 37°C with shaking at 220 rpm. A 1 ml sample of culture was diluted 3-fold every 2 h and the optical density at 600 nm (OD_600_) was measured using a T6 UV spectrophotometer using a Purkinje General Instrument (Beijing, China) to plot the growth curve. Cells were harvested by centrifugation at 10,000 *×* g after culturing for 12 h, washed three times with sterile water, and diluted 100-fold for morphological assay. A 3 μl sample of cell diluent was placed in the centre of a 13 mm × 18 mm piece of mica, freeze-dried for 8 h, and observed by scanning electron microscopy (SEM) using a Zeiss Merlin Compact instrument (Jena, Thuringia**,** Germany). Cells for atomic force microscopy (AFM) analysis must be washed repeatedly and diluted at appropriate times. A sample of 3–5 μl was placed on a 25 mm × 25 mm fresh, sterile mica sheet and freeze-dried for 8 h before analysis by a Bruker ICON instrument (Berlin, Germany).

We selected the ScanAsyst-air scanning mode to image cells of each strain. For the probe, a silicon tip on a nitride lever was employed, with a cantilever length of 115 μm and a light spring constant of 0.4 N/m. The morphology of colonies on LB agar plates was observed by stereo microscopy using a Motic str6 instrument (Xiamen, China) with a silicon tip on a nitride lever.

### Analysis of Extracellular Pigments and Proteins

The supernatants of LG3145 and WT strains were collected from LB medium cultures by centrifugation (6,000 *×* g, 20 min, 4°C) and mixed with precooled 12.5% trichloroacetic acid. Following freezing (−20°C for 12 h), the supernatant was centrifuged at 4°C for 5 min to collect proteins. The precipitate was washed 3–5 times with 80% precooled acetone to remove residual trichloroacetic acid and stood for 30 min to completely evaporate acetone. Some precipitates were dialysed for 5 h and freeze-dried for 8 h, then observed using an Oxford X-max20 energy dispersive spectrometer (EDS) and by SEM; other precipitates were dissolved in 1 ml ddH_2_O and mixed with 5 ml Folin-phenol reagent A and 0.5 ml reagent B, both purchased from Lanji technology (Shanghai, China). After standing at ambient temperature for 30 min, the mixture was analyzed using a spectrophotometer at 700 nm (Wan et al., 2009; Han et al., 2019). The protein concentration was calculated using the equation y = 0.2823x + 0.1011 (*y*-axis = absorbance, *x*-axis = protein concentration).

For pigment analysis, minimal medium (MM; 3.48 g KH_2_PO_4_, 1.5 g Na_2_HPO_4_.12H_2_O, 3.96 g (NH_4_)_2_SO_4_, 0.7 g MgSO_4_.7H_2_O, 0.01 g yeast extract, 1 L ddH^2^O, pH 7–7.5) supplemented with 2.5% (w/v) glycerol or glucose was used to culture each strain at 35°C with shaking at 220 rpm. The absorbance of extracellular pigments was measured from 200 to 800 nm by a Shimadzu UV-3600plus spectrophotometer (Kyoto, Japan). All experiments were repeated in triplicate.

## Results and Discussion

### UgRNA:Cas9 Edits Multiple *cre* Sites

The *cre* sites of seven target genes were edited by transforming UgRNA:Cas9 expression plasmids pCas9-*Cax*, pCas9-*Cac* and pCas9-*Cpt* into WT strain protoplast following the strategy shown in Figure 2. Chloromycetin (25 μg/ml) was added to the CMR media used for selecting transformants harbouring the UgRNA:Cas9 system.

The promoter regions, 500 bp upstream fragments of target genes *ackA*, *ntdA*, *ptsH*, *budA*, *acsA*, *xylA* and *sucC*, were amplified by PCR (Supplementary Figure S1) and mutations of the seven target genes were confirmed by DNA sequencing (Supplementary Figure S2). Surprisingly, there were eight mutants with the same genotype among the 10 transformants (success rate = 80%).

The mutation maps of each gene (top of Supplementary Figure S2) show that all point mutations occurred around the target fragments, and the closer the mutations to the centre, the denser they are, and most are distributed along the direction from PAM to target fragment. The number of mutations was significantly positively correlated with the identity of UgRNAs shown in Table 1 (except for *xylA*). Most of the mutations are transitions, with almost no deletions. Thus, a UgRNA:Cas9 system with more than 50% identity can edit multiple genes at the same time. In fact, the genomes of the mutants were determined by next-generation sequencing (NGS) and the results indicate that most of the mutation sites occurred around the seven target fragments, but some mutation sites occurred at other sites. The occurrence of off-target sites editing appeared to correlate with the similarities of UgRNAs. These relationships will be investigated in future work.

**TABLE 1:** Mutation type analysis of target genes.

Gene	*ackA*	*ntdA*	*acsA*	*ptsH*	*budA*	*xylA*	*sucC*
Identity	70%	70%	55%	55%	50%	50%	30%
Mutations	16	14	10	15	10	28	5
Transversion	8	5	1	2	7	10	4
Transition	8	9	9	13	4	17	1
Deletion						1	

According to editing characteristics, the DNA strand around the target may be twisted and rolled, especially the DNA strand in the binding direction of UgRNAs, and the higher the similarity between UgRNA and target fragment, the larger the curl range. Because the UgRNAs were partially complementary to the target fragments, Cas9 endonuclease seems to wobble within this region and recognise multiple PAM sites for cleaving, which can be verified by the locations and the corresponding PAMs of mutation points shown in Table 2. The results indicate that all mutations occurred in the promoters, which include 1–2 bp mutations at target fragments according to the preset PAM, especially the edited sites of *ptsH*, *acsA* and *sucC* which coincided exactly with *cre* sites. There were many off-target mutation points with an efficient PAM nearby, such as the transversion T > A(A > T) near the -10 domain of *ackA*, with a CAG after10 nt; the transversions T > A and A > T of *bud*A, also with TGG and AGG after two and five nucleotides respectively; the point mutation T > G(A > C) of *ntdA* with a CAG after 2 nt; and the mutation C > T before *cre* of *xylA* with an efficient PAM ‘TAG’. All data are marked with a grey shadow in Table 2. However, there were a few distinct off-target mutations in *suc*C, and only one base exactly edited at the target fragment, possibly because the lower the similarity of UgRNAs, the fewer the number of DNA fragments involved. This phenomenon further proves that UgRNA:Cas9 may cause swing editing of Cas9, and the swing range is affected by UgRNA identity.

**TABLE 2:** Mutation analysis of target genes.

Gene	Identity (%)	Mutation type	PAM	Mutation sites		Annotation
				Target fragment^a^	PAM^b^	
*ackA*	70	CA > TG	AGG	0	–−2,–3	
		T > A(A > T)	CAG	–32	–10	
*ntdA*	70	G > T	CAG	0	–20	complementary PAM
		G > T		–6	–26	
		T > G(A > C)	CAG	+25	–2	
*ptsH*	55	T > C	TTG	0	–7	
		A > T(T > A)		+11	+8	complementary PAM
		A > T	AGG	+80	–8	
*budA*	55	A > T	TGG	–23	–50	
		T > A	TGG	–32	–2	
		A > T	AGG	+162	–5	
*acsA*	50	G > T	AGG	0	–12	
		A > G		0	–23	
*xylA*	50	T > A	AGG	0	–4	
		C > T	TAG	–10	–2	
*sucC*	30	A > *n*(T > *n*)	GCG	0	–1	complementary PAM
		T > A(A > T)		0	–13	

^a^Distance from target fragment; ^b^Distance from PAM.

### UgRNA:Cas9 Mediates Carbon Catabolism

The DNA sequencing analysis described above showed that multiple *cre* sites of *B. pumilus* LG3145 were edited successfully. Additionally, various phenotypes of LG3145 were markedly altered, including resistance to sugar, capsules outside cells, and white flocculent protein floating on the medium upper layer. We subsequently analysed changes in carbon catabolism, and explored the relationships between mutations and phenotypes.

#### Peptidoglycan Synthesis is Enhanced in *B. pumilus* LG3145

**FIGURE 3:**
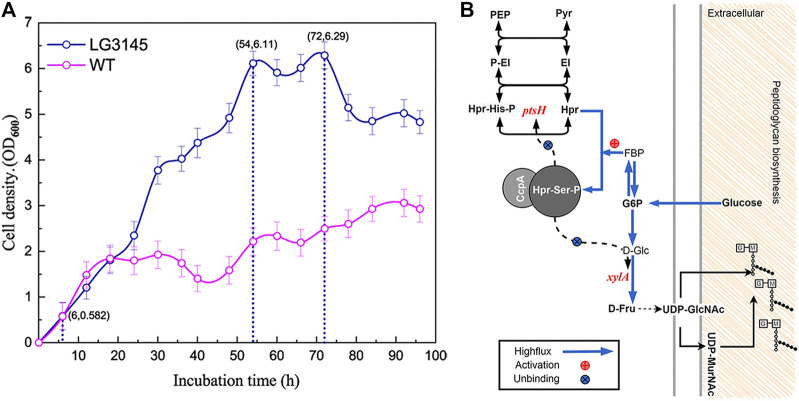
**(A)** Comparison of the growth patterns of LG3145 mutant and WT cells. Periodic (2 h) optical density (OD) readings were taken to assess cell growth of strains in GYN plus 30% (w/v) glucose at 37°C with shaking at 220 rpm. **(B)** Peptidoglycan synthesis-related pathways.

In *Bacillus*, raising the amount of carbon *in vivo* can lead to carbon catabolite repression (CCR) and inhibit the absorption of glucose. Herein, we compared the growth patterns of the WT and the multiple *cre* editing LG3145 strain in high sugar broth. The results indicate that LG3145 cells were strongly resistant to sugar after editing around the *cre* site (Figure 3A). LG3145 cells entered a vigorous exponential growth phase (lasting ∼48 h) after 6 h of cultivation, during which the cell density (OD_600_) was >6, whereas the WT strain displayed a shorter (14 h) exponential growth phase with an OD_600_ < 2. Furthermore, AFM images revealed a large number of secretions outside the bacteria, believed to be bacterial glycocalyx, that help to protect against environmental stresses (Su et al., 2012; Meng et al., 2017) (Supplementary Figure S3C). The glycocalyx outside LG3145 cells may be related to the level of glucose catabolites because when we used 10% glucose GYN the glycocalyx became slimmer and flimsier (Supplementary Figure S3A). Even in LB broth, we observed a thicker polysaccharide capsule outside the cells of the LG3145 strain in TEM and AFM images (Supplementary Figures S4B, S4C, black arrow) compared with WT cells.

To further demonstrate this phenomenon, traditional negative staining using Indian ink and safranin was used to observe the presence of capsules by microscopy, and halos clearly surrounded each LG3145 cell, as expected (Supplementary Figure S4A). We speculate that the main components of the capsule are peptidoglycan because *cre* site mutation of *xylA* encoding xylose isomerase resulted in the CcpA∼(Hpr-Ser-p) complex that was unable to bind its promoter, regardless of how much glucose was supplied, and xylose isomerase was expressed normally (Dahl and Hillen, 1995). Additionally, the *ptsH* gene, encoding the key HPr factor associated with CCR, was also mutated at the *cre* site. In the LG3145 strain, more glucose was transported into cells, and expression of Hpr and Hpr-Ser-p was elevated, but this did not repress *ptsH* and *xylA* (Figure 3B) (Kraus et al., 1994). Thus, when glucose was increased, D-Fru was produced in greater quantities, the flux from D-Fru to UDP-GlcNac and UDP-MurNAc was enhanced, and more peptidoglycan was synthesised and secreted from cells to form the capsules that protect cells against dehydration in high-permeability glucose medium (Lovering et al., 2012). This explains why LG3145 cells could survive better than WT cells (Supplementary Figure S3B).

**FIGURE 4:**
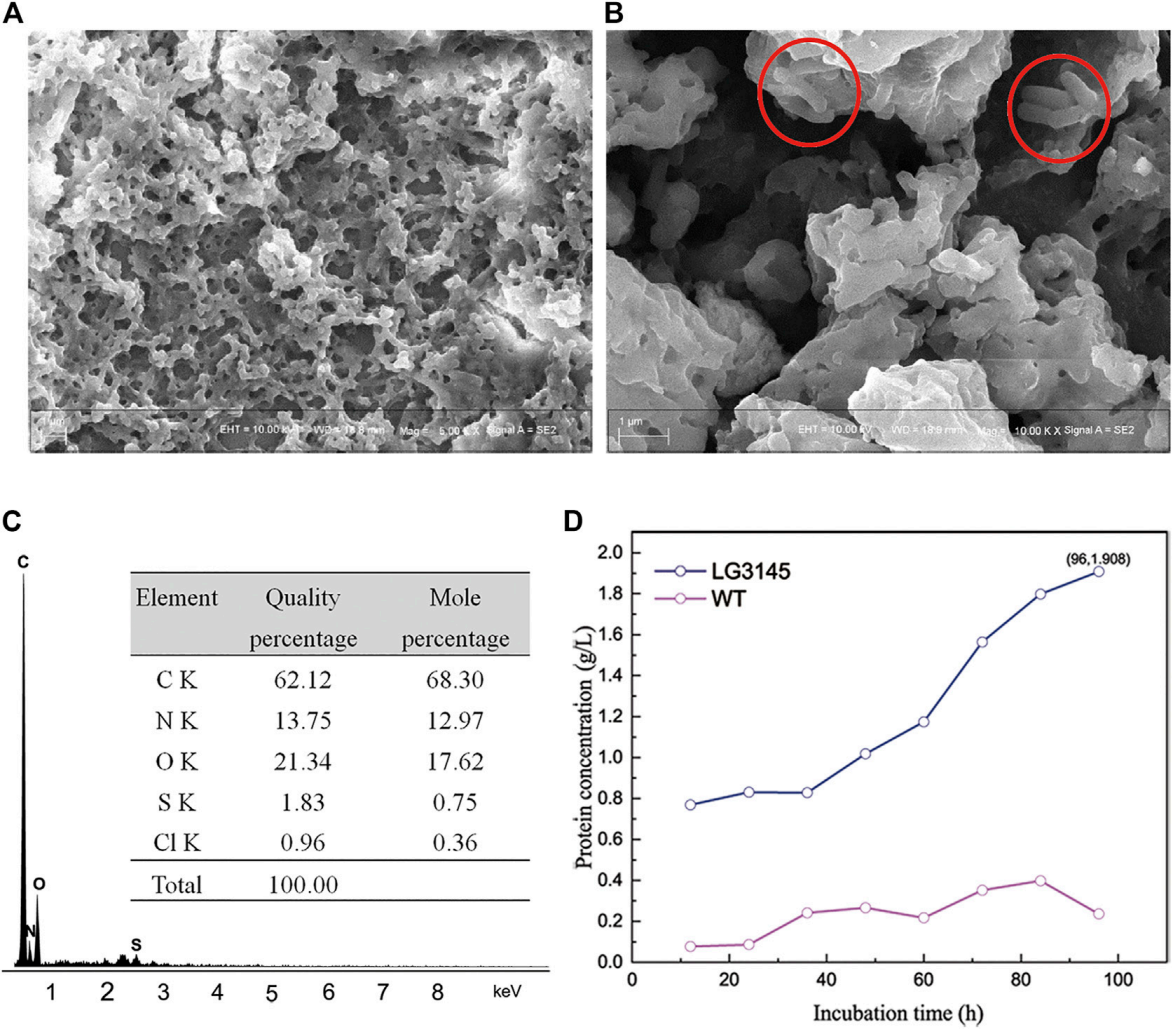
**(A)** Extracellular protein analysis and **(B)** detailed SEM images. **(C)** Sample energy dispersive spectrum (EDS) showing the elemental ratios. **(D)** Comparison of extracellular protein concentrations between LG3145 and WT cells.

The glycocalyx is believed to be made from polypeptide, polysaccharide, or both, and secreted from cells (Li et al., 2018; Scarff et al., 2018; Rausch et al., 2019; Sun and Zhang, 2021). Coincidentally, we observed a white flocculent biofilm on the surface of the medium when LG3145 cells were incubated in a test tube (Supplementary Figure S5B). The biofilm substance was isolated by dialysis and observed by SEM, which revealed porous and viscous properties of protein (Figure 4A). When culturing, we could see that some cells were embedded (red circle in Figure 4B). The biofilm substance was found to contain C, N, O and S elements, the main elements of amino acids (Figure 4C), according to the results of EDS analysis. Total extracellular protein levels reached 1.908 g/L after 96 h, as determined by the Folin-Phenol method, but there was little protein *in vitro* with the WT strain (Figure 4D). Protease activity was also assessed using a two-layer skim-milk plate (Supplementary Figure S5C). This indicated that editing *cre* sites caused an increase in extracellular proteolytic activity at the late exponential phase. All experiments were repeated in triplicate and the same phenomenon was observed for LG3145.

#### Cytochrome Synthesis is Enhanced in *B. pumilus* LG3145

**FIGURE 5:**
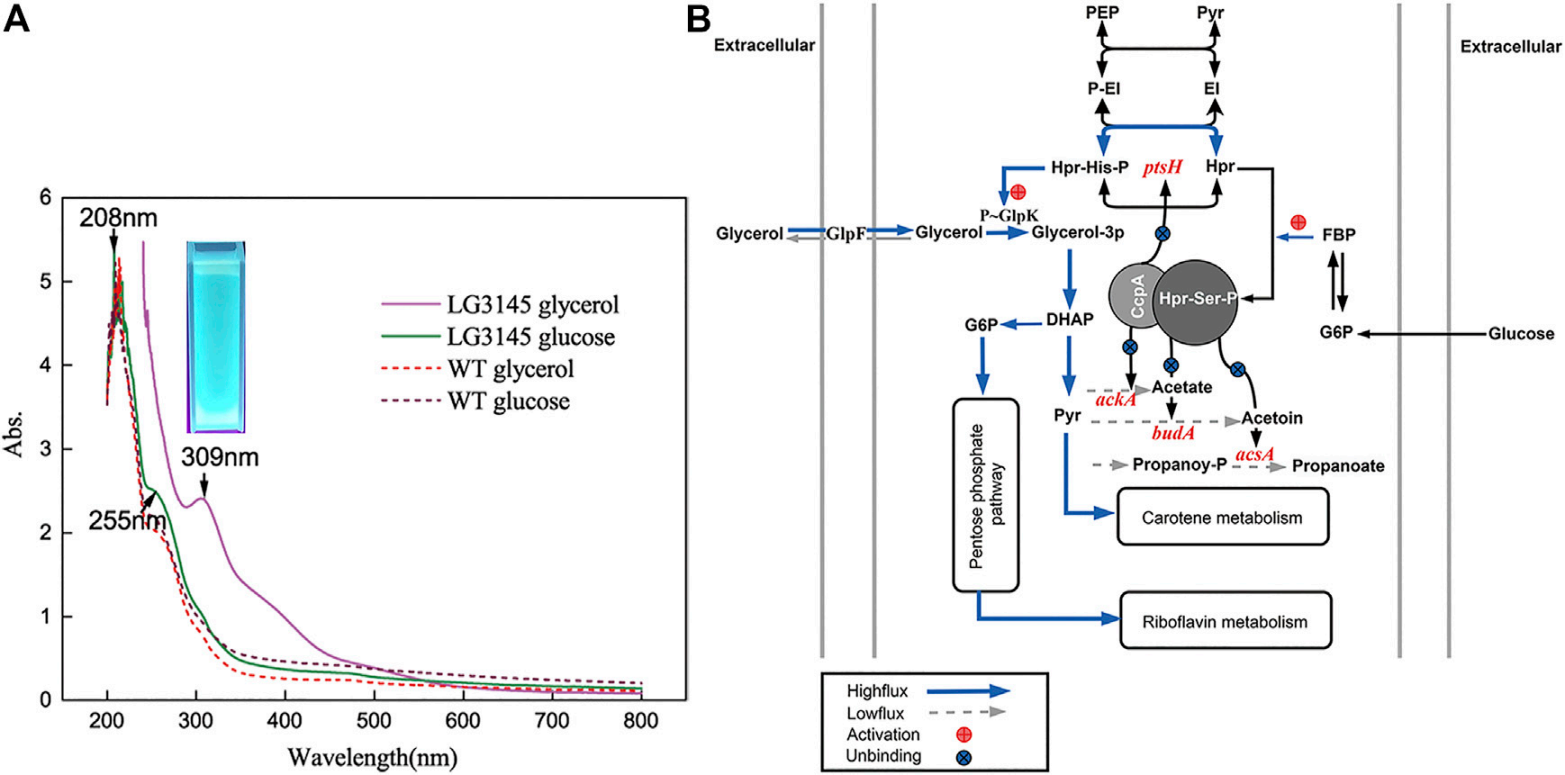
**(A)** Cytochrome synthesis-related pathways. **(B)** UV-Vis spectral analysis of LG3145 and WT cells incubated in MM supplemented with glycerol or glucose and cultured at 37°C. Images were obtained using a Chemidoc XRS + system (*trans*-UV 302 nm, BioRad, United States).

Since previous studies on CCR in *Bacillus* showed that *cre* negatively regulates genes related to carbohydrate catabolism (Sousa et al., 2019), mutation of *cre* could potentially disrupt secondary pathways, and lead to a significant increase in extracellular excretion. When we incubated the mutant LG3145 strain on LB agar, colonies were more adhesive and pigmented under the naked eye, with fuzzy edges, whereas WT colonies appeared smooth, white, and with distinct edges (Supplementary Figures S6A, 6B). To further explore the relationship between pigmentation and carbon source, MM with different carbon sources was used to culture each strain. Amazingly, an orange-coloured suspension of LG3145 cells was evident on day 3 in MM supplemented with 2.5% (w/v) glycerol or glucose after culturing at 35°C. In particular, with glycerol as the carbon source, the pigmentation of the suspension was more intense than with the preferred carbon source (glucose), while the broth of WT cells was colourless (Supplementary Figures S6C, 6D). These results imply that mutation of *cre* sites of target genes disrupted the carbon catabolism network, specifically by increasing the flux of secondary pathways, when utilising carbon sources other than glucose (Chen et al., 2019). As shown in Figure 5A, *cre* site mutation of *ptsH* resulted in overexpression of the Hpr protein, and the amount of Hpr-His-p and Hpr-Ser-p was also increased. Glycerol as a carbon source is transported by the transporter GlpF, then converted into glycerol-3-phosphate by glycerol kinase GlpK. The activity of GlpK is induced by Hpr-P-His through the promotion of GlpK phosphorylation. Thus, in LG3145 cells, more Hpr-His-P was produced, more GlpK was converted to p-GlpK, and more glycerol-3-phosphate was present in cells. In the next step, glycerol-3-phosphate is converted to dihydroxyacetone phosphate (DHAP), which enters the glycolytic pathway, and the flux from glycerol to the glycolytic pathway is enhanced following mutation of *ptsH*. However, the overflow pathways from pyruvate to acetate, acetoin and propanoate were repressed, as shown in Figure 5A, because *cre* site mutation of *ackA*, *acsA* and *budA* must be activated by CcpA∼Hpr-Ser-p complex binding to *cre* sites, which differs from the CCR mechanism of other genes (Turinsky et al., 2000; Moir-Blais et al., 2001). Thus, glycerol metabolic flux is diverted to the pentose phosphate pathway or to secondary metabolism, and pigments such as riboflavin or carotene accumulate, resulting in a colour change. The results of UV-Vis spectral analysis revealed maximal peaks shifting from 255 to 309 nm when LG3145 cells were cultured with glycerol as a carbon source (Figure 5B), and the culture emitted 450–480 nm blue-green phosphorescence upon UV excitation. The profiles resembled the features indicative of some cytochromes, but the molecular organisation of the pigments remains to be determined in future studies.

#### Secondary Metabolism is Enhanced in *B. pumilus* LG3145

**FIGURE 6:**
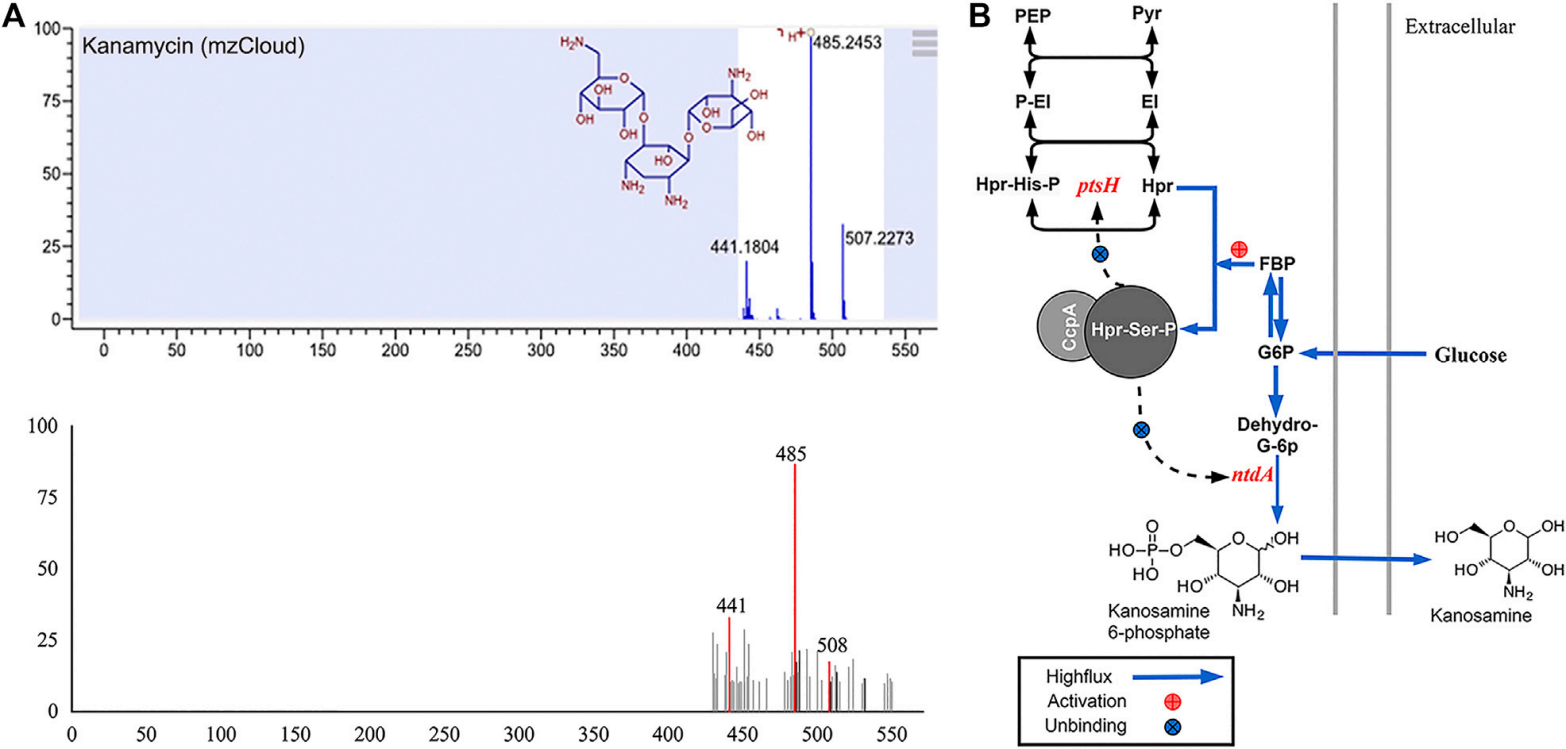
**(A)** MS spectrum showing kanamycin among LG3145 metabolites and the standard spectrum in mzCloud. **(B)** Kanosamine synthesis-related pathways in *B. pumilus*.

For cytochrome experiments, liquid chromatography-mass spectrometry (LC-MS) was employed to probe molecular organisation. Unexpectedly, we found an amino sugar antibiotic, kanosamine (3-amino-3-deoxy-D-glucose), in the extracellular secretions of LG3145 cells incubated in MM supplemented with glucose. The mass spectrum of the metabolite (Figure 6A) resembled the kanamycin standard spectrum in the mzCloud MS database. Kanosamine, a component of kanamycin, can be produced in a three-step pathway from glucose 6-phosphate in *Bacillus spp.* (Prasertanan and Palmer, 2019).

As shown in Figure 6B, glucose is phosphorylated after entering cells, and the more glucose 6-phosphate, the more CcpA∼(Hpr-Ser-p) complex *in vivo*. However, in LG3145, *cre* site mutation at *ntdA* encoding a sugar aminotransferase (Van Straaten et al., 2013) resulted in the CcpA∼(Hpr-Ser-p) complex that was unable to bind the promoter, regardless of how much glucose was included in the medium. Additionally, *ptsH* encoding the key HPr factor related to CCR, was also mutated at the *cre* site, and the more glucose transported into the cells, the greater the expression of Hpr and Hpr-Ser-p, but this did not repress expression of *ptsH* and *ntdA*. When glucose is increased, the kanosamine synthesis pathway is enhanced and secretion is elevated.

## Conclusion

Herein, a novel CRISPR gene editing strategy was used to edit multi-target *cre*-boxes and relieve CCR in *B. pumilus*. We constructed a series of unspecific gRNAs as crRNAs according to similarity in the CRISPR/Cas9 system. Using seven metabolic genes as the editing targets, wobble editing occurred around *cre* sites, and this positively correlated with similarity. In addition, the phenotypes of the LG3145 mutant strain were markedly altered, and this was correlated with the seven edited genes. These phenotypes demonstrated that mutation at multiple *cre* sites caused target gene transcription changes and disruption of the carbon central pathway, which resulted in an increase in secondary metabolites such as extracellular pigments, proteins, and kanosamine, and the formation of polysaccharide capsules. The *B. pumilus* LG3145 strain has great potential as a host organism for secretion in cell factory processes.

## Data Availability

The datasets presented in this study can be found in online repositories. The names of the repository/repositories and accession number(s) can be found in the article/Supplementary Material.
