# Supplementary material for: Wobble Editing of Cre-box by Unspecific CRISPR/Cas9 Causes CCR Release and Phenotypic Changes in Bacillus pumilus
Source: Front Chem. 2021 Aug 9;9:717609. doi: 10.3389/fchem.2021.717609 (PMC8381255; doi:10.3389/fchem.2021.717609)
Supplement: Supplementary file 1 [file DataSheet1.pdf]

TABLE S1 *Cre box* sequence in genes

Gene		*cre box* sequence	Distance (bp) to:	
			TxSSa	TISSb
ackA	acetate kinase	ATGTAAGCGGTA ACA	-52	+165
ntdA	aminotransferase	TTGCCAATGCTGCGT	-34	+3525
ptsH	phosphocarrier protein HPr	TGGCCAATGGATCAA	-113	+937
budA	acetolactate decarboxylase	ATGAAAACAAATCT	+189	+341
acsA	acetyl-CoA synthetase	GTGGAAACGCTACCA	-70	-6
xylA	xylose isomerase	AAGGAATCGGAAAAA	-307	-38
sucC	succinate-CoA ligase[ADP-forming] subunit beta	ATGAAAGCGCAGTCT	-6	+42
	**consensus sequencesc**	**WWGNAANCGNWNNCW**		

a. TxSS, transcriptional start site.
b. TISS, translational start site.
c. W:A /T N:A/T/G/C

TABLE S2 List of UgRNAs used in this study

UgRNA	Target sequence*	Array of UgRNA	Identity	PAM	Gene
Cac	TGT AAGCGGTAACAGTTCAA	TGTAAGCGTTATCAATACGC	70%	AGG	*ackA*
Cpt	CGCACCAAATGCGCCAGACG	CGCAACCAAAGCTTCAGATT	70%	CAG	*ntdA*
	AAGCCACCAAGCAGTTCTGT		55%	TTG	*ptsH*
	CAAATCTCGTTCACCTTGTT		50%	TGG	*budA*
Cax	GTGGAAACGCTACCATCAGT	GAAAGCGTTACCAGCAATAG	55%	AGG	*acsA*
	ATCGGAAAAATACAATTTGA		50%	AGG	*xylA*
	CAAAAAATATAAAATAGACT		30%	GCG	*sucC*

*Target sequence design website http://crispor.tefor.net/

TABLE S3 Strains used in this study

Strain	Characteristics	Source (Usage)
Escherichia coli top10	Host for plasmid construction, F⁻ mcrA Δ(mrr-hsdRMS-mcrBC) φ80 lacZΔM15Δ lacX74 recA1 araΔ139Δ(ara-leu)7697 galUgalK rpsL (StrR) endA1 nupG	Lab stock
Escherichia coli DH5α	Host for pCas9	Lab stock
Bacillus pumilus WT	Gene sequencing is the same as *Bacillus pumilus* SH-B9	Soil
	**Gene editing**	
Bacillus pumilus LG3145	*Bacillus pumilus* WT derivate	This work

TABLE S4 Plasmids used in this study

Plasmid	Characteristics	Source
pCas9	Bacterial expression of Cas9 nuclease, tracrRNA and crRNA guide, *E. coli*	Addgene http://www.addgene.org/42876/
	**Gene editing**	
pCas9-*Cac*	pCas9 derivate, containing crRNA similarity with *cre*-box of *ack*A	This work
pCas9-*Cpt*	pCas9 derivate, containing crRNA similarity with *cre*-boxes of *ptsH*, *ntdA* and *ackA*	This work
pCas9-*Cax*	pCas9 derivate, containing crRNA similarity with *cre*-boxes of *acsA*, *xylA* and *sucC*	This work

TABLE S5 Primers used in this study

Primers	Sequence	Template or Description
	**Array of gRNA for cre-box wobble editing**	
Cac-F	AAACTGTAAGCGTTATCAATACGC	annealed to gRNA Cac
Cac-R	AAAAGCGTATTGATAACGCTTACA	
Cpt-F	AAACCGCAACCAAAGCTTCAGATT	annealed to gRNA Cat
Cpt -R	AAAAAATCTGAAGCTTTGGTTGCG	
Cax-F	AAACGAAAGCGTTACCAGCAATAG	annealed to gRNA Cax
Cax-R	AAAACTATTGCTGGTAACGCTTTC	
	**Primers for PCR**	
ackA-F	AAAAAGCACGCCAAAAGTTTGTTTGTG	used for colony *ackA*
ackA-R	TTTCAACAAGACCTTTTGTTAAAACGGTT	
ntdA -F	TCATCATGGCGGTGTGGCTCTATAA	used for colony *ntdA*
ntdA -R	GCATAGCTTCCACTCACAACAAGCA	
ptsH-F	GCGATTTACCATGAAGCAAAACCAAAGA	used for colony *ptsH*
ptsH-R	ACACGTAAACGCGTAATACAAGCATCA	
budA-F	CACGTGTGATATCGGGTCTCACG	used for colony *budA*
budA-R	CTTCTGGCCATTTTTGACTGGCG	
acsA-F	GCCTTCATGAAATGTATAGTTCGCAAGC	used for colony *acsA*
acsA-R	TCTGAACGTATACTTTTCATCCCGCTG	
xylA-F	TGCATCTTGTAGCGTTGTTT	used for colony *xylA*
xylA-R	CAATGTCACGGTCATGGAAC	
sucC-F	CCGTAATTTAGTCGAACTCATGCGC	used for colony *sucC*
sucC-R	TCTTTTGTTTTTGCAATTTTTACCCCGC	

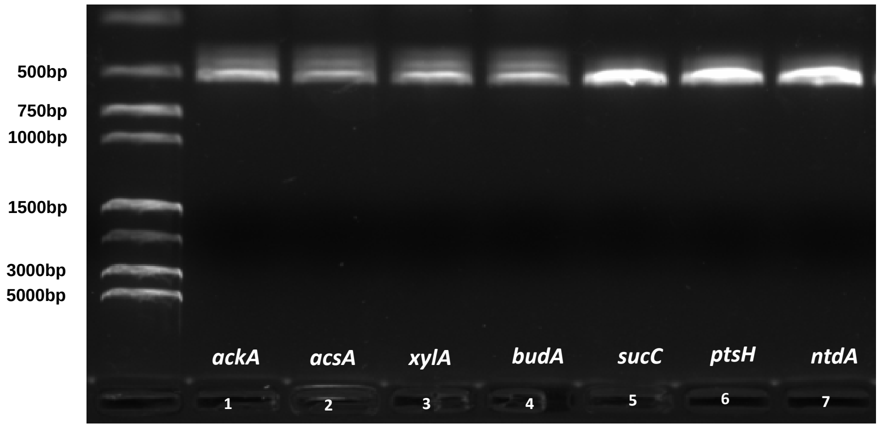

FIGURE S1 Agarose gel electrophoresis analysis of PCR production.
M, DNA markers; 1, *ackA*; 2, *acsA*; 3, *xylA*; 4, *budA*; 5, *sucC*; 6, *ptsH*; 7, *ntdA*.

ackA UP12_RS13195

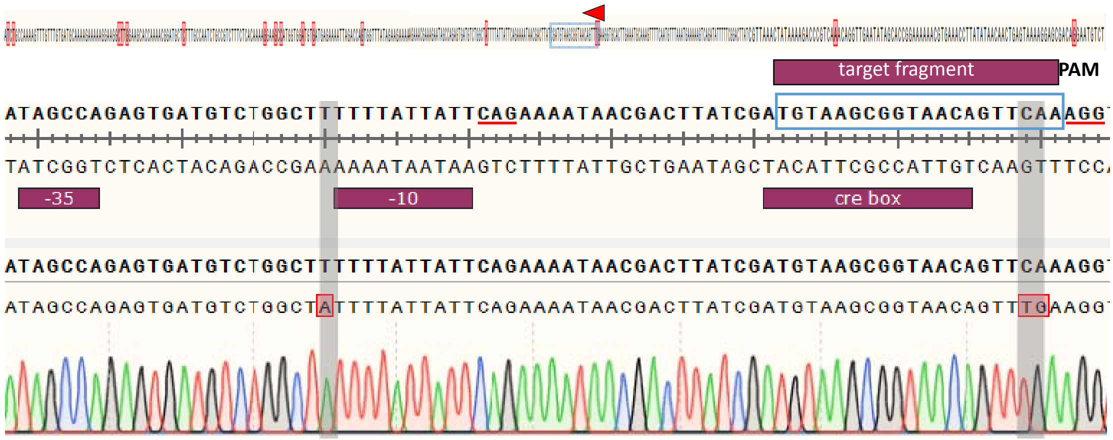

ntdA UP12_RS01730

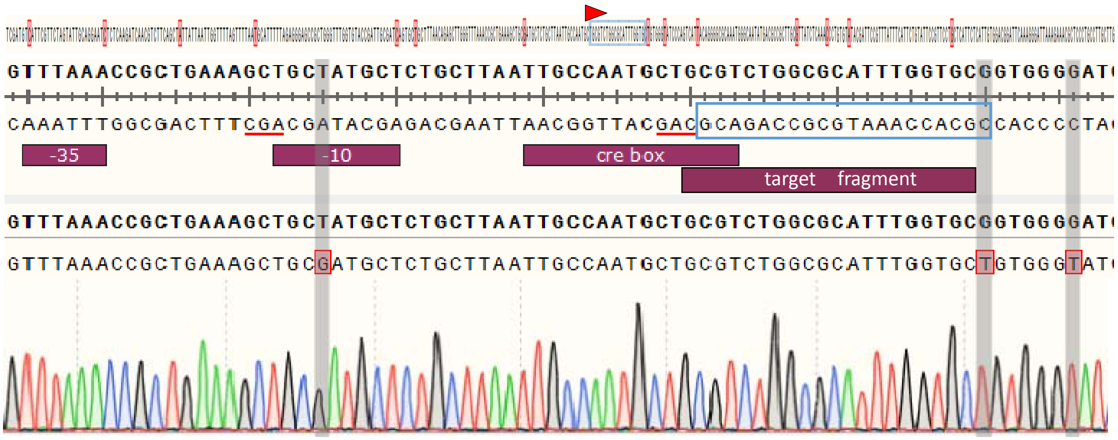

ptsH UP12_RS06700

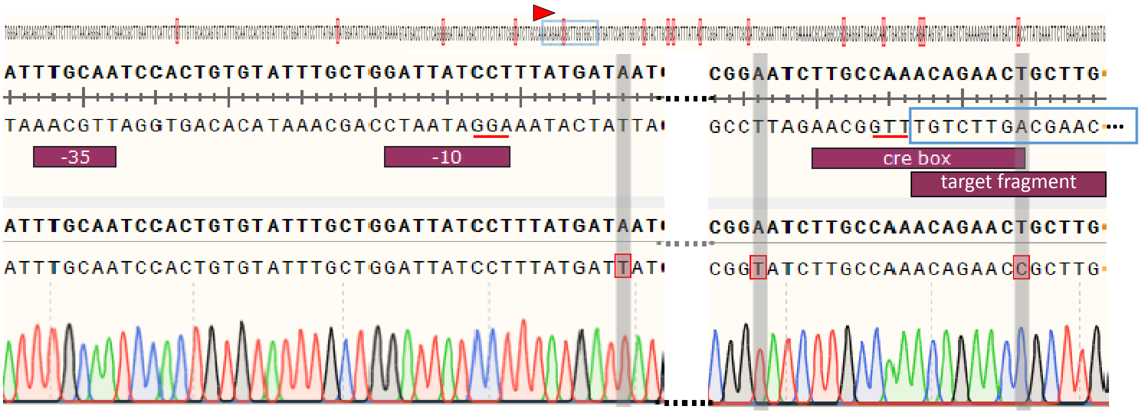

budA UP12_RS16915

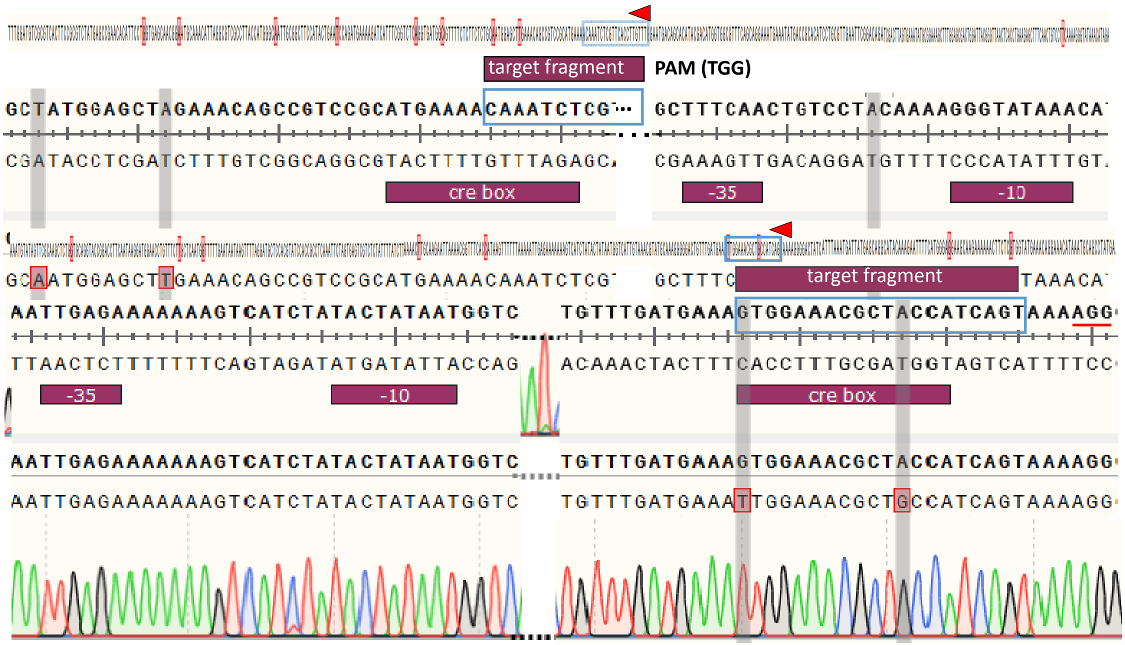

xylA UP12_RS09365

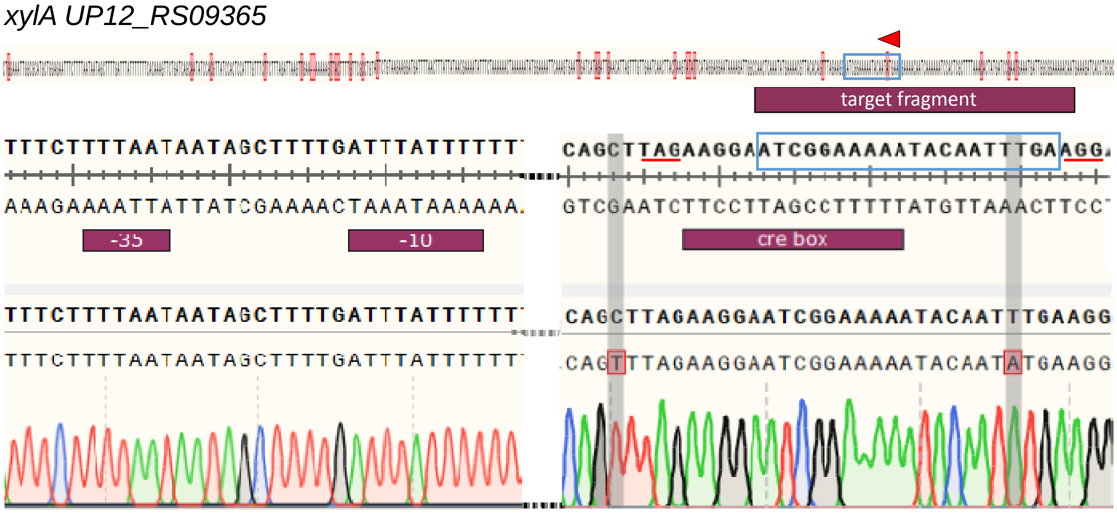

sucC UP12_RS07835

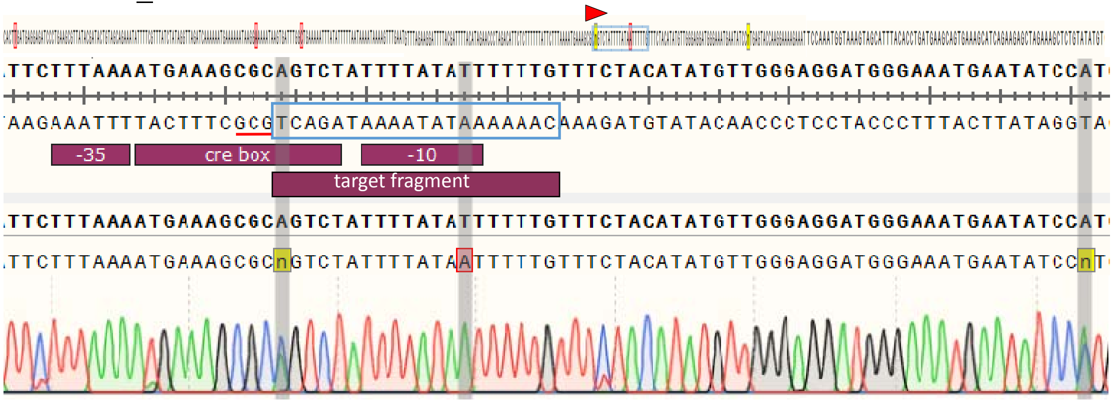

FIGURE S2 Chromatograms of Sanger sequencing of edited *cre* sites of seven genes in *B. pumilus* LG3145. ► = PAM position; ▮ = distinct mutation bases; n = indistinct mutation bases.

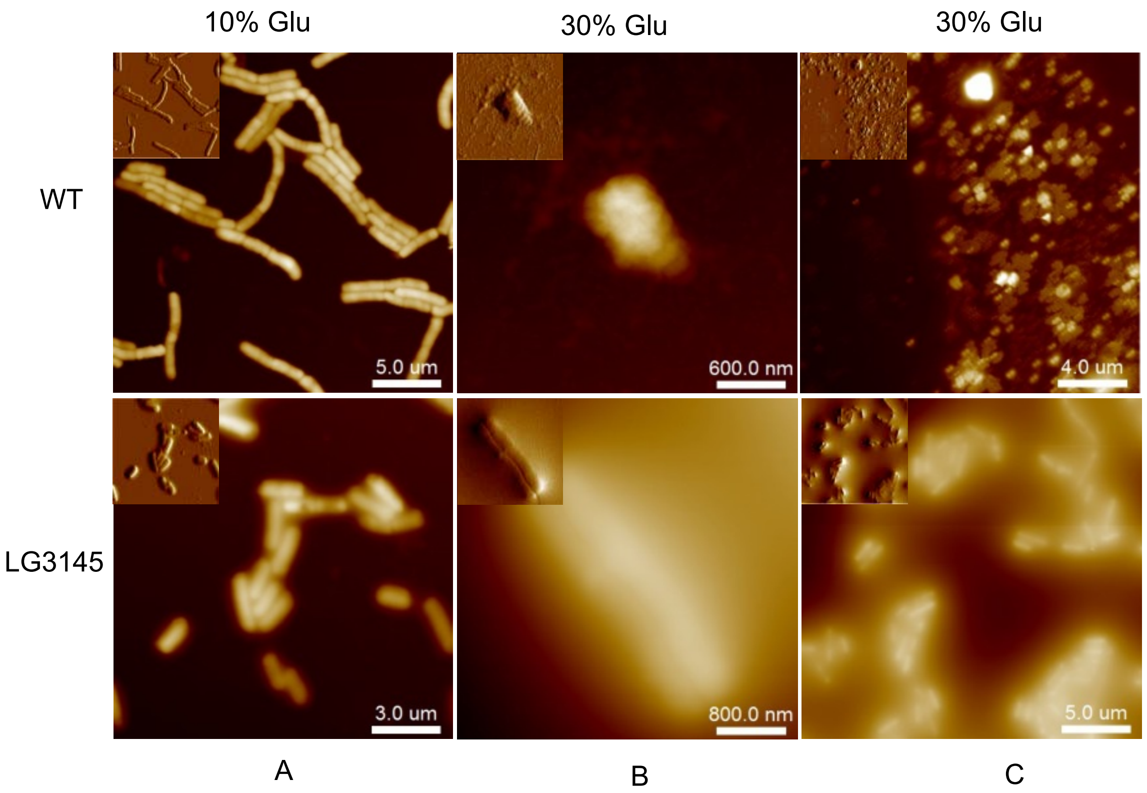

FIGURE S3 AFM phase images of WT and LG3145 cells with error images on the top-left. (A) Capsulated LG3145 cells imaged by AFM when incubated with 10% glucose, and images of WT cells with clear edges. (B) Amplitude image of WT single cells showing partial disappearance when incubated in 30% glucose medium, compared with LG3145 cells coated with capsules. (C) Images of multiple WT cells which have mostly been lysed, and LG3145 cells enveloped in a thick glycocalyx, which are normal and strong when grown in 30% glucose medium.

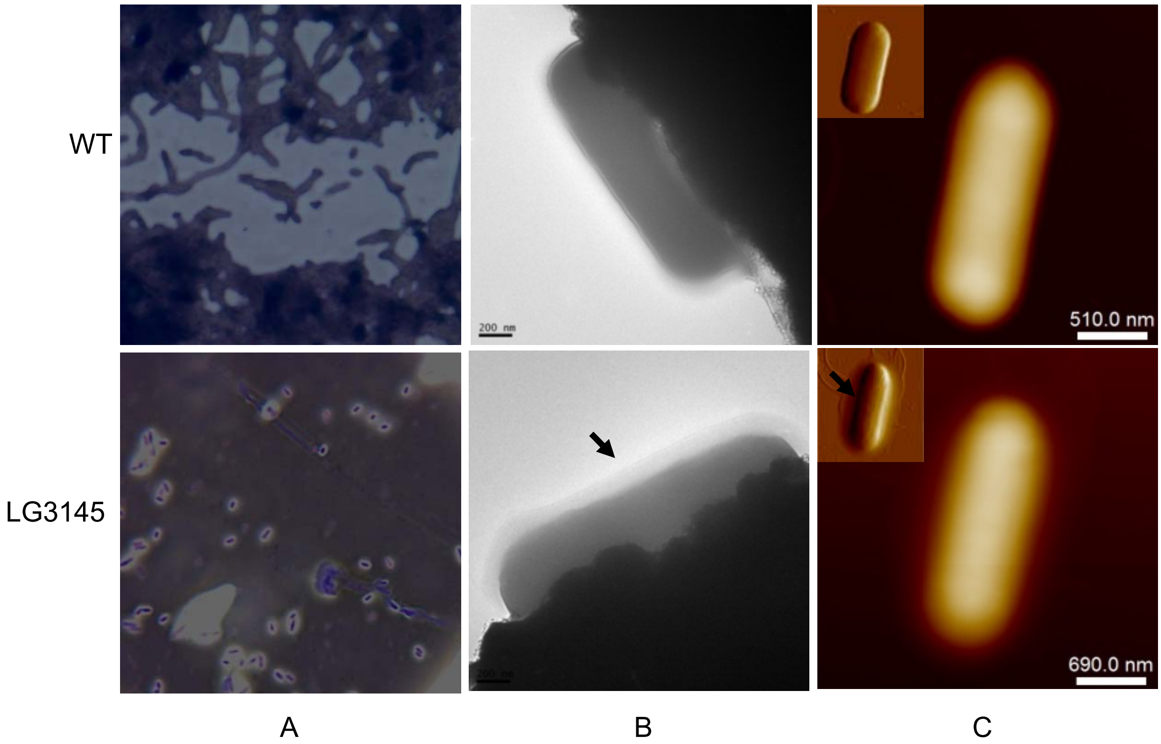

FIGURE S4 Analysis of WT and LG3145 cells in LB broth following negative staining (A), TEM (B), and AFM (C). Images of a LG3145 cells show a sheet structure capsule, but WT cells lack this structure.

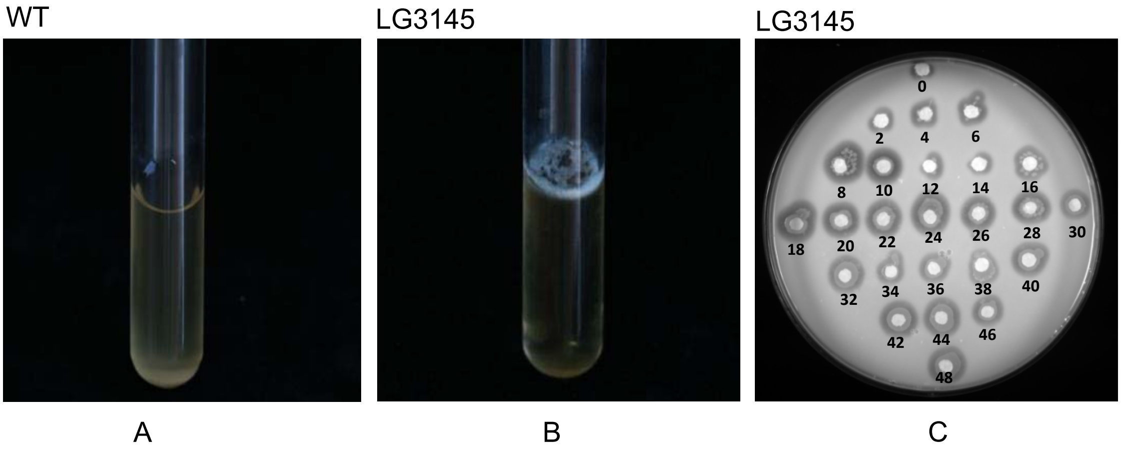

FIGURE S5 Phenotypes of WT (A) and LG3145 (B) cells in terms of biofilm production in LB medium, and a LG3145 protein activity map generated using the skim milk plate method (C).

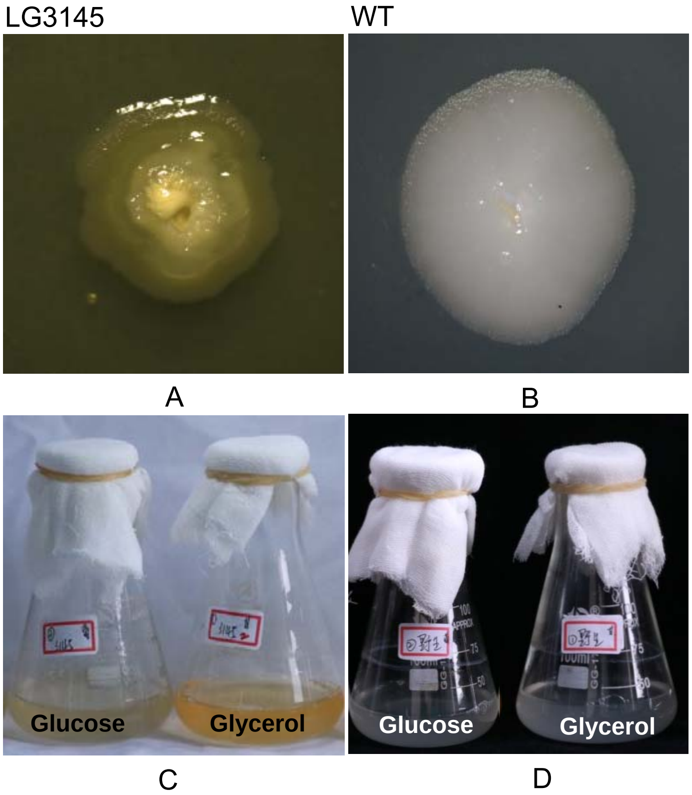

FIGURE S6 Morphology of colonies of (A) LG3145 and (B) WT cells grown on LB agar (24 h, 37°C). Pigmentation was observed after 3 days of culture for (C) LG3145 and (D) WT cells grown in MM plus glycerin and glucose, respectively (2.5%w/v, 37°C, 225 rpm).
